# The facts of the matter: What is a hormone?

**DOI:** 10.1371/journal.pgen.1008938

**Published:** 2020-06-26

**Authors:** Gerard Karsenty

**Affiliations:** Department of Genetics and Development, Columbia University Irving Medical Center, New York, New York, United States of America; HudsonAlpha Institute for Biotechnology, UNITED STATES

By choice, scientists value facts over beliefs. This is why science is constantly moving forward; it embraces novelty, provided it is fortified by evidence and all sorts of controls, checks and balances, as a sign of and a tool for progress.

A goal common to all individuals that devote their life to science is to advance knowledge. They may have other goals but this particular one has to be common to all scientists. This goal also creates a burden and not such an easy one to bear—the burden of restraint. Scientists live under the public light: they publish, i.e., share their findings with the scientific community at large and sometimes with the general public. What comes with that are huge responsibilities, the one to be right of course, but more importantly the responsibility to be cautious, to stay within the data and to not drag them beyond what they actually show. Otherwise the pursuit of knowledge is, unwillingly but definitely, tainted. Results have to be controlled over and over, and of course, just as importantly, their presentation has to be neutral as opposed to clever. This serves as a necessary preamble to a series of facts.

The word hormone means “that which sets in motion”, and was first used in 1902 before the emergence of molecular biology, yet its carefully worded definition has essentially remained unchanged. This definition is at the heart of this piece and our work on the endocrinology of bone over the past 13 years.

To be clear, a hormone is defined both experimentally and physiologically by *what it does when present in abundance in animals or in humans* because of an environmental stimulus, physiological perturbation, tumor, genetic abnormality, or when injected in an otherwise normal human being or animal of any species. As long as a molecule has not been shown to affect a particular physiological function when injected in a normal animal, it may be many things, it may have many names but a hormone it may not be. I cite here a recent and vivid example of what I write. Mice lacking the GLP1 receptor have a mild metabolic phenotype at best, yet GLP1 plays a critical role in physiology and its analogs are best-selling drugs for the treatment of diabetes [1]. Likewise, the blossoming field of FGF21 biology is based on gain of function experiments. What I write is not meant to disparage mouse genetics since it is part of what my lab has used to address many questions in the field of bone biology and I have learnt to appreciate its value. It is rather meant to remind myself and everyone else that biology is an assembly of disciplines and approaches. Scientists are not and should not be children in a toy store. The newest tool does not, and is not meant to, replace the older one, rather it enriches the tool kit. Even more to the point, one experimental approach with very small N, based on erroneous protocols, using mixed genetic backgrounds, does not suffice to invalidate, even if carefully worded, the work done by many others using multiple and state-of-the-art experimental approaches.

I write the preceding to introduce another fact: osteocalcin was shown to have properties of a hormone in mice, rats, monkeys and subsequently humans through gain-of-function experiments [2–52]. Those experiments occupy more than half of the original paper [50]. It was the analysis of mice generated for another purpose, but that happened to have too much osteocalcin that set this field in motion. These mice, the Esp-/- mice (*Esp* is a gene preferentially expressed in osteoblasts), were not only hyper-osteocalcinemic they were also hyperinsulinemic, hypertestosteronemic, agitated, and had the ability to run much longer than their wild type littermates [50,53]. Granted, this could have had many causes, but one of them that deserved to be tested was whether it was explained in part by their increased circulating osteocalcin levels. Thereafter it was only by 1) realizing that the phenotypes of *Esp-/-* and *Osteocalcin-/-* mice were mirror images of each other 2) performing genetic complementation experiments and 3) injecting osteocalcin in wild type mice or treating cells with osteocalcin that the notion that osteocalcin was a hormone was advanced [6,50,53–55].

I stand by these results unequivocally. I stand by them because they were so unexpected by us and everybody else that reviewers, rightly so, asked for control after control, each of which made the results more robust. The results were more robust because of the large number of mice (N over 50 by now in both sexes for every experiment). They were also more robust because they used cross-validating assays, every genetic control, different genetic backgrounds, both sexes, and various ages. They were more robust, and more credible, because for each experiment we used gold-standard techniques exactly as described by experts in fields other than our own. In fact, in most cases these experiments were conducted not by us, but by labs with whom we had shared the *Ocn-/-* or *Esp-/-* or other relevant mutant mice and who were world experts in the particular assays being conducted. To name just a very small portion, euglycemic clamps were done in the laboratory of Jason Kim, islet perifusion in the lab of Klaus Kaestner, indirect calorimetry in the lab of Jeffrey Pessin, conditional fear conditioning in the lab of Rene Hen, electron micrographs of testes in the one of Louis Hermo, whole cell electrophysiology in the lab of Xiao-Bing Gao, and *in vivo* nerve fiber recordings in the lab of Kamal Rahmouni. These are, by all criteria, renowned experts in their respective fields and there are many more that cannot be listed here. Together they brought histology, electron microscopy, electrophysiology, indirect calorimetry and metabolomics evidence to the picture. I repeat, by our own cautious design, each of these scientists worked independently with full latitude to verify or contradict our results and it is thanks to their work that our results are so robust. Thirteen years later, even if it was painful at the time, I take this opportunity to thank each and every reviewer for having made this work what it is.

Since the initial publication proposing that osteocalcin is a hormone there have been hundreds of publications confirming that osteocalcin behaves as a hormone that signals through two specific receptors that were notably identified by two competing groups and that elicits particular physiological responses in mice and humans. These studies, through different *in vivo* and *in vitro* assays, all show that osteocalcin, and by that I mean bioactive (undercarboxylated) and not total osteocalcin, has the properties of a hormone. These publications come from investigators across many fields; investigators I know, investigators I do not know, and I have never interacted with and even, investigators that were fierce competitors in making discoveries about the endocrine functions of osteocalcin. Some of these publications are from prominent investigators or a Nobel laureate, others from less recognized but equally hard-working, truth-seeking scientists who were not scared off or were perhaps attracted by novelty. Where the papers were published is irrelevant because, in my view, science is not driven by impact factor but by the rigorous, controlled, verified, pursuit of knowledge, novel knowledge. What is rewarding as an endocrinologist is to see that a large portion of this body of work was done as it should be, through gain-of-function experiments, and part of it in human cells or patients [9,39,51,52,56–61]. These publications come from over 30 groups and counting, across 5 continents, they were reviewed at journals by colleagues who were not necessarily benevolent, revised, re-reviewed, and finally published. As such they earned their credibility and deserve the respect that goes to any scientific publication. Not more respect of course, but certainly (why would it be?) not less. A few of them are listed below [2–49,52]. Taken together, over the past 13 years, these papers form a body of work, the very beginnings of a field. This near unanimity (science is a human activity after all and there is never complete unanimity) despite using different hands, models and assays is what more than anything else supports the idea that osteocalcin is a hormone. Let’s be absolutely clear, even if there were 1000 of these publications, the sheer number them would not be enough to make them right. But to prove them wrong the very same experiments from body weight measurements, to electron microscopy, and electrophysiology, etc. have to be performed in the same conditions and achieve different results, otherwise we are not speaking of scientific facts.

In all fairness, and I’m well aware of it, for every 20 publications or so showing that osteocalcin is a hormone there was always one failing to do so. Often there are technical or biological reasons for the discrepancy and the reason has often taught us new things about this new hormone whose nature we are only beginning to uncover. For example, one of them uses an outbred CRISPR generated, but not sequenced, rat model with N of 3 to 5 per group per experiment. Others may have measured total (as opposed to undercarboxylated) osteocalcin in humans, which makes it hard to rigorously determine the endocrinological relevance because these levels do not necessarily correlate with levels of bioactive osteocalcin. Those discrepancies and others have often served as reminders that this new hormone will likely be fertile territory for discovery for many years to come.

Incidentally, none of them, until now, came with an editorial [62] declaring that the *book is closed* and osteocalcin is not a hormone for *the rest of time* and insinuating (for most readers I know) scientific incompetence or superficiality on my part. I personally do not know of a scientific book that is ever *closed*. But *PLOS Genetics* just published 2 papers that contradict each other on many points but claim that 2 mouse models lacking osteocalcin are more or less wild type. They are accompanied with a vivid perspective, cleverly written that unquestionably raises doubt on the way I conduct science.

If a hormone is defined by what it does when in excess of baseline in normal animals, then unlike what the Perspective claims from its first to its last paragraph, neither of these papers addresses the question of whether osteocalcin is a hormone. They did not inject wild–type animals with any of the many preparations of osteocalcin shown by many other studies to work [2–49]. They simply tested whether the mice they generated and analyzed, through protocols different from those used by mainstream investigators, do not have a phenotype that we described in mice we generated and analyzed through different protocols. As such they join the small but respectable contingent of people who do not believe that osteocalcin is a hormone and do not believe bone is an endocrine organ. There is nothing wrong with that view *a priori*. It becomes wrong when these two papers are presented as the final truth. Although I will not address in detail whether or not there are experimental flaws in these studies, I will note that in the published reviews one of the reviewers commented that the genetic backgrounds, standard deviations and the number of animals analyzed per experiment were all questionable. The reviewer also mentioned that the conclusions were “too sweeping” and should be corrected.

Besides overt qualitative and quantitative differences in the ways the assays were performed the reasons for the discrepancies between their results and those of 30 labs is for now unknown and I am fully aware of it. But such discrepancies are the way novel discoveries, especially those effecting human health, often occur. The answer to this question can only emerge by exchanging mice, performing controls, analyzing them extensively, using the complete panel of appropriate endocrine tests and verifying comprehensively the outcome and specificity of the genetic methodologies used to inactivate *Osteocalcin*.

I need to state here that the *Ocn-/-* mice we generated have been and are available on demand and are already found all over the world. Moreover, these mutant mice have also been deposited at, and are available from, the Jackson Laboratories (https://www.jax.org/strain/034070). In contrast, and despite several solemn pronouncements in their papers to journalists and in social media for about a year, mice from the other parties have not been deposited at the Jackson Laboratories at the time I write this article. This is why I have contacted Dr. Williams who acknowledged that indeed his mouse model has not been submitted to the Jackson Laboratories. We are working diligently with him to exchange mice.

In addition to this exchange of reagents that I hope will occur for the sake of the entire community there is another question that scientists and journalists alike have asked me. If the papers published in *PLOS Genetics* have not addressed the definition of a hormone, why would their conclusions and a Perspective written by one of their reviewers, who is an osteoporosis expert, attack, with ardor, the work of the many labs who have addressed this question in mice, rats, monkeys and humans? I am not the one who can answer this question.

Science does not work through inference. Novelty comes through hard work, through the use of the entire experimental gamut, control after control, literally I would say through blood, sweat (a lot of it) and tears (some). Before telling hundreds of different scientists from different fields that they are all wrong, have always been, that their results are serving a dogma proposed by a slow-witted scientist that burdens federal funds one must conduct thoughtful, exhaustive and robust experimentation. I have been asked, often by the same people, after each of my contributions to the field: “Runx2 does not control bone formation”, “central neuronal control of bone mass is a fantasy”, “serotonin does not regulate bone mass”, “bone mass is not coordinated with energy metabolism hormones and reproduction” and now “osteocalcin is not a hormone”. None of these questions or their accompanying papers stood the test of time. Science is not politics and there is no room in science for what has the appearance of veiled character assassination. Before saying that a single novel truth based on negative and incomplete data has emerged overnight, one needs to do more than simply say it, one needs to walk the walk and demonstrate it. This was not done. Do these results discredit, unwillingly or not, previous work? In the short-term, with some people, maybe; but scientific life is much longer than that.
